# Supplementary material for: Circulation of a Unique Klebsiella pneumoniae Clone, ST147 NDM-1/OXA-48, in Two Diverse Hospitals in Calabria (Italy)
Source: Antibiotics (Basel). 2025 Jan 26;14(2):128. doi: 10.3390/antibiotics14020128 (PMC11851497; doi:10.3390/antibiotics14020128)
Supplement: Supplementary file 1 [file antibiotics-14-00128-s001.zip › antibiotics-3387050-supplementary.pdf]

Sample	β-lactams									Fluoroquinolones	Aminoglycosides		Colistin
	CAZ	AMC	TZP	FEP	CZA	MEM	MEV	IMI	AZT	CIP	AK	CN	COL
Breakpoint	S≤1<br>R>4	S≤8<br>R>8	S≤8<br>R>8	S≤1<br>R>4	S≤8<br>R>8	S≤2<br>R>8	S≤8<br>R>8	S≤2<br>R>4	S≤1<br>R>4	S≤0.25<br>R>0.5	S≤8<br>R>8	S≤2<br>R>2	S≤2<br>R>2
CS01	>256 R	>256 R	>256 R	>256 R	>256 R	64 R	192 R	> 32 R	>1024 R	>32 R	>256 R	>256 R	1 S
CS02	>256 R	>256 R	>256 R	>256 R	>256 R	96 R	256 R	> 32 R	>1024 R	>32 R	>256 R	>256 R	0,75 S
CS03	>256 R	>256 R	>256 R	>256 R	>256 R	48 R	32 R	> 32 R	>1024 R	24 R	>256 R	>256 R	0,75 S
CS04	>256 R	>256 R	>256 R	>256 R	>256 R	32 R	32 R	> 32 R	>1024 R	>32 R	>256 R	>256 R	1 S
CS05	>256 R	>256 R	>256 R	>256 R	>256 R	64 R	>256 R	> 32 R	>1024 R	>32 R	>256 R	>256 R	12 R
CS06	>256 R	>256 R	>256 R	>256 R	>256 R	48 R	24 R	> 32 R	>1024 R	24 R	>256 R	>256 R	1 S
CS07	>256 R	>256 R	>256 R	>256 R	>256 R	64 R	128 R	> 32 R	>1024 R	32 R	>256 R	>256 R	8 R
CS08	>256 R	>256 R	>256 R	>256 R	>256 R	96 R	64 R	> 32 R	>1024 R	24 R	>256 R	>256 R	16 R
CS09	>256 R	>256 R	>256 R	>256 R	>256 R	96 R	48 R	> 32 R	>1024 R	>32 R	>256 R	>256 R	12 R
CS10	>256 R	>256 R	>256 R	>256 R	>256 R	96 R	48 R	> 32 R	>1024 R	>32 R	>256 R	>256 R	4 R
CS11	>256 R	>256 R	>256 R	>256 R	>256 R	64 R	32 R	> 32 R	>1024 R	>32 R	>256 R	>256 R	1 S
CS12	>256 R	>256 R	>256 R	>256 R	>256 R	64 R	32 R	> 32 R	>1024 R	>32 R	>256 R	>256 R	1,5 S
CS13	>256 R	>256 R	>256 R	>256 R	>256 R	64 R	32 R	> 32 R	>1024 R	>32 R	>256 R	>256 R	8 R
CS14	>256 R	>256 R	>256 R	>256 R	>256 R	64 R	>256 R	> 32 R	>1024 R	>32 R	>256 R	>256 R	16 R
CS15	>256 R	>256 R	>256 R	16 R	16 R	0,064 S	0,064 S	0,094 S	>1024 R	>32 R	>256 R	>256 R	1 S
CS16	>256 R	>256 R	>256 R	>256 R	>256 R	64 R	32 R	> 32 R	>1024 R	>32 R	>256 R	>256 R	24 R
CS17	>256 R	>256 R	>256 R	>256 R	>256 R	48 R	24 R	8 R	>1024 R	>32 R	>256 R	>256 R	1 S
CS18	>256 R	>256 R	>256 R	>256 R	48 R	0,5 S	4 S	0,75 S	>1024 R	>32 R	>256 R	>256 R	1 S
CS19	>256 R	>256 R	>256 R	>256 R	>256 R	64 R	64 R	> 32 R	>1024 R	>32 R	>256 R	>256 R	16 R
CS20	>256 R	>256 R	>256 R	>256 R	>256 R	32 R	16 R	8 R	>1024 R	>32 R	>256 R	>256 R	0,75 S
CS21	>256 R	>256 R	>256 R	>256 R	>256 R	48 R	24 R	> 32 R	>1024 R	>32 R	>256 R	>256 R	1,5 S
CS22	>256 R	>256 R	>256 R	>256 R	>256 R	64 R	32 R	> 32 R	>1024 R	>32 R	>256 R	>256 R	1,5 S
CS23	>256 R	>256 R	>256 R	>256 R	>256 R	96 R	96	> 32 R	>1024 R	>32 R	>256 R	>256 R	12 R
CS24	>256 R	>256 R	>256 R	>256 R	>256 R	96 R	24 R	> 32 R	>1024 R	>32 R	>256 R	>256 R	3 R
CS25	>256 R	>256 R	>256 R	>256 R	>256 R	32 R	32 R	> 32 R	>1024 R	>32 R	>256 R	>256 R	1 S
CS26	>256 R	>256 R	>256 R	>256 R	>256 R	48 R	128 R	> 32 R	>1024 R	>32 R	>256 R	>256 R	1 S
CS27	>256 R	>256 R	>256 R	>256 R	>256 R	64 R	24 R	24 R	>1024 R	>32 R	>256 R	>256 R	1 S
CS28	>256 R	>256 R	>256 R	>256 R	>256 R	128 R	96 R	> 32 R	>1024 R	>32 R	>256 R	>256 R	8 R
CZ01	>256 R	>256 R	>256 R	>256 R	16 R	96 R	32R	> 32 R	>1024 R	>32 R	>256 R	>256 R	1 S
CZ02	>256 R	>256 R	>256 R	>256 R	48 R	64 R	32 R	> 32 R	>1024 R	>32 R	>256 R	>256 R	1,5 S
CZ03	>256 R	>256 R	>256 R	>256 R	48 R	64 R	256 R	> 32 R	>1024 R	>32 R	>256 R	>256 R	1 S
CZ04	>256 R	>256 R	>256 R	>256 R	>256 R	64 R	32 R	> 32 R	>1024 R	>32 R	>256 R	>256 R	1 S
CZ05	>256 R	>256 R	>256 R	>256 R	48 R	64 R	32 R	> 32 R	>1024 R	>32 R	>256 R	>256 R	1 S
CZ06	>256 R	>256 R	>256 R	>256 R	48 R	64 R	48 R	> 32 R	>1024 R	>32 R	>256 R	>256 R	1 S
CZ07	>256 R	>256 R	>256 R	>256 R	>256 R	64 R	32 R	> 32 R	>1024 R	>32 R	>256 R	>256 R	0,75 S
CZ08	>256 R	>256 R	>256 R	>256 R	48 R	64 R	32 R	> 32 R	>1024 R	>32 R	>256 R	>256 R	1 S
CZ09	>256 R	>256 R	>256 R	>256 R	48 R	64 R	24 R	> 32 R	>1024 R	>32 R	>256 R	>256 R	0,75 S
CZ10	>256 R	>256 R	>256 R	>256 R	>256 R	96 R	32 R	> 32 R	>1024 R	>32 R	>256 R	>256 R	1 S
CZ11	>256 R	>256 R	>256 R	>256 R	>256 R	64 R	48 R	> 32 R	>1024 R	>32 R	>256 R	>256 R	0,75 S
CZ12	>256 R	>256 R	>256 R	>256 R	>256 R	64 R	32 R	> 32 R	>1024 R	>32 R	>256 R	>256 R	1,5 S
CZ13	>256 R	>256 R	>256 R	>256 R	>256 R	96 R	32 R	> 32 R	>1024 R	>32 R	>256 R	>256 R	< 0.5 S
CZ14	>256 R	>256 R	>256 R	>256 R	>256 R	64 R	48 R	> 32 R	>1024 R	>32 R	>256 R	>256 R	< 0.5 S
CZ15	>256 R	>256 R	>256 R	>256 R	>256 R	64 R	32 R	> 32 R	>1024 R	>32 R	>256 R	>256 R	< 0.5 S
CZ16	>256 R	>256 R	>256 R	>256 R	48 R	64 R	24 R	> 32 R	>1024 R	>32 R	>256 R	>256 R	< 0.5 S
CZ17	>256 R	>256 R	>256 R	>256 R	4 S	64 R	1 S	> 32 R	>1024 R	>32 R	>256 R	>256 R	< 0.5 S
CZ18	>256 R	>256 R	>256 R	>256 R	>256 R	64 R	>256 R	> 32 R	>1024 R	>32 R	>256 R	>256 R	< 0.5 S
CZ19	>256 R	>256 R	>256 R	>256 R	>256 R	64 R	32 R	> 32 R	>1024 R	>32 R	>256 R	>256 R	< 0.5 S
CZ20	>256 R	>256 R	>256 R	>256 R	>256 R	96 R	32 R	> 32 R	>1024 R	>32 R	>256 R	>256 R	< 0.5 S
CZ21	>256 R	>256 R	>256 R	>256 R	48 R	64 R	>256 R	> 32 R	>1024 R	>32 R	>256 R	>256 R	< 0.5 S

TableS1, the table shows the resistance/susceptibility profiles of the analized strains for the most important tested antibiotic Molecules: ceftazidime (CAZ), amoxicilline/clavulanate (AMC), piperacilline/tazobactam (TZP), cefepime (FEP), ceftazidime/avibactam (CZA), meropenem (MEM), meropenem/vaborbactam (MEV), imipenem (IMI), aztreonam (AZT), ciprofloxacin (CIP), amikacin (AK), gentamicin (CN), colistin (COL). Resistant strain (R), susceptible strain (S). Antibiotic breakpoints of clinical isolates were interpreted according to EUCAST v.13.1, 2023 guidelines for interpretation of MICs.
